# iPro2L-Kresidual: A High-Performance Promoter Identification Model for Sequence Nonlinearity and Context Mining

**DOI:** 10.3390/genes16121412

**Published:** 2025-11-27

**Authors:** Yanjuan Li, Shicai Li, Guojun Sheng, Yu Chen

**Affiliations:** 1 College of Electrical and Information Engineering, Quzhou University, Quzhou 324000, China; 2 College of Computer and Control Engineering, Northeast Forestry University, Harbin 150040, China

**Keywords:** DNA promoter, deep learning, two-stage prediction, bioinformatics, sequence analysis

## Abstract

A promoter is an important non-coding DNA sequence, as it can regulate gene expression. Its abnormalities are closely associated with various diseases, such as coronary heart disease, diabetes, and tumors. Therefore, promoter identification is highly significant. Due to the insufficient nonlinear feature extraction and insufficient capture of sequence context relationships, existing single promoter identification models have a lower classification performance. To overcome these shortcomings, this paper proposed a new model called iPro2L-Kresidual. iPro2L-Kresidual integrated a residual structure with a KAN network to design a novel Kresidual module. The Kresidual module significantly enhanced the nonlinear expression capability of sequence features by using B-spline functions and residual networks. Additionally, to fully capture the sequence context relationship, iPro2L-Kresidual improved a Transformer encoder module by replacing the linear processing method with gated recurrent units, so then it can extract both local and global context features of a sequence. Furthermore, iPro2L-Kresidual designed a regularized label smoothing cross-entropy loss function to ensure training stability and prevent the model from becoming overly confident. Experimental results on 5-fold cross-validation showed that the accuracy of promoter identification and promoter strength identification, respectively, was 94.28% and 90.55%. Moreover, on an independent dataset, the prediction accuracy reached 93.13%, further demonstrating the model’s strong generalization ability. This provides a novel and effective predictive model for promoter site prediction.

## 1. Introduction

A promoter is a specific DNA sequence located upstream of the 5′ end of a structural gene. Its function is to activate RNA polymerase, thereby enabling the enzyme to bind accurately to the template DNA and initiate transcription with specificity. At the same time, the promoter acts as a particular segment of DNA within an organism, functioning like a “switch” that controls gene regulation and expression. For promoters regulating gene expression, the promoter identification is of great significance, especially for understanding diseases caused by gene mutations, such as cancer and coronary heart disease.

However, with the explosive growth of genetic data, the cost of traditional manual screening and identification of promoters has significantly increased. It not only requires a large amount of manpower but also takes a considerable amount of time, making it an extremely inefficient method for researchers. Some promoter identification studies are developed based on sequence alignment. Sequence alignment methods that match the gap fingerprints of unannotated sequences with those of annotated sequences [1,2] are both labor-intensive and time-consuming. Similarly, some approaches that treat promoter-related elements—such as the TATA box and CCAAT box—as predictive signals [3] ignore the non-element portions of the sequence, and they often result in poor predictive performance. In addition, content-based approaches take into account the frequency distribution of k-mer fragments and focus on regions with a high frequency of CpG sites [4]. CpGProD [5], CONPRO [6], and Eponin [7] are some signal-based and content-based methods for eukaryotic promoter prediction. However, the adjustment of sequence alignment is entirely dependent on the systematic use of reference sequences, which reduces performance when dealing with distantly related sequence sets [8]. In addition, due to their heuristic nature and the high memory requirements for aligning longer sequences, sequence alignment methods tend to perform relatively poorly [9]. ConSite [10], PromH [11], and FootPrinter [12] are also some studies of promoter prediction based on sequence alignment. In general, due to the limitations of sequence alignment algorithms and the complexity of promoter sequences, the performance of promoter identification based on sequence alignment is relatively poor.

To improve the performance of promoter identification, researchers have proposed a series of computational approaches. Yanrong Ji et al. [13] introduced the DNABERT model. The model converts DNA sequences into k-mer tokens as input, with each k-mer representing a segment of nucleotide sequence. These sequences are then further represented in matrix form, where each k-mer token is embedded into a numerical vector, thus transforming biological information into a mathematical representation that the model can process. In addition, during training, a multi-head self-attention mechanism is employed, enabling the model to capture contextual relationships among different parts of the sequence, thereby allowing for more accurate prediction of biological functions such as gene expression regulation. Qingwen Li et al. [14] proposed an attention-based method using long short-term memory (LSTM). This approach utilizes the k-mer technique for word segmentation and feature extraction, employs Word2Vec to train word embeddings, and uses both SVM and attention-based LSTM models for prediction. Zhimin Zhang et al. [15] proposed the iPromoter-CLA model, an innovative hybrid deep learning framework that combines capsule networks with bidirectional long short-term memory (Bi-LSTM). The model first processes DNA sequences using one-hot encoding, and then employs a one-dimensional convolutional layer, a capsule network layer, and a Bi-LSTM layer to learn both local and global features of the sequence. Finally, a self-attention mechanism is applied to select relevant features, thereby improving prediction accuracy and enabling effective prediction of promoters and their strength. Huijuan Qiao et al. [16] proposed the iPro-GAN model, which extracts features from DNA sequences by employing a spatiotemporal self-cross-correlation method based on the Moran coefficient combined with the physicochemical properties of dinucleotides. The extracted features are then classified using a deep convolutional generative adversarial network (GAN-DC). Aliraza et al. [17] proposed the iPro-TCN model, which incorporates the word2vec algorithm and temporal convolutional networks (TCNs). In this approach, DNA sequences are represented as language models, with each nucleotide considered as a “word.” The word2vec method is then used to convert the sequences into feature matrices, and the temporal convolutional network is employed to process the sequential data, specifically designed for handling one-dimensional data with a temporal dimension. Binchao Peng et al. [18] proposed the iProL model, which utilizes a pre-trained Longformer model to embed DNA sequences as text and obtain DNA embedding vectors. Features are then extracted using CNN and BiLSTM, while LIME and STREME tools are applied to analyze the interpretability of the model’s predictions, ultimately resulting in more accurate prediction outcomes. Yazi Li et al. [19] proposed the msBERT-Promoter model, which is based on the BERT pre-trained model. The approach segments the original sequences into tokens of varying lengths using different tokenization strategies, thereby generating distinct feature matrices. These are then fed into the BERT layers to extract latent information among sequences. Finally, a soft voting ensemble method is used to combine the predictions of different base models.

This paper proposed the iPro2L-Kresidual model, which combines multi-scale convolution, the Kresidual module, an improved Transformer encoder, and parallel pooling to identify promoters. The iPro2L-Kresidual outperforms state-of-the-art models on public datasets. In the experimental validation for promoter identification, the accuracy reached 94.28%, while in the experiment for determining promoter strength, the accuracy reached 90.55%. The accuracy in independent dataset validation reached 93.13%. This provides a new and effective tool for the promoter prediction task.

The main contributions of this study are as follows:

(1) The present study uses hybrid encoding of one-hot and NCP to represent promoter sequences, extracts features using multi-scale convolution and the Kresidual module. Multi-scale convolution effectively captures multi-scale local features within the sequence, while Kresidual module can learn nonlinear features by using the nonlinear representation ability of the KAN network and strengthening the expression of key features of residual mechanism.
(2) We use an improved Transformer encoder architecture to address the insufficient capture of context relationships during promoter recognition. The linear processing module in the Transformer encoder is replaced with a Gated Recurrent Unit (GRU) to model the context relationships between features comprehensively. The GRU, with its unique structure, captures local context information within the sequence, while the Transformer encoder can capture long-range dependencies, thereby understanding the global context of the sequence. The effective combination of these two components enhances the accuracy of promoter identification.
(3) We design an improved loss function, namely, the regularized label smoothing cross-entropy loss function. The loss function effectively solves training instability and overconfidence of the model, and the problem of neglecting the similarity between promoter categories.
(4) This work designs three types of experiments, including ablation experiments, benchmark dataset testing, and independent dataset testing, to comprehensively validate the performance of the iPro2L-Kresidual model.

## 2. Materials and Methods

**Figure 1:**
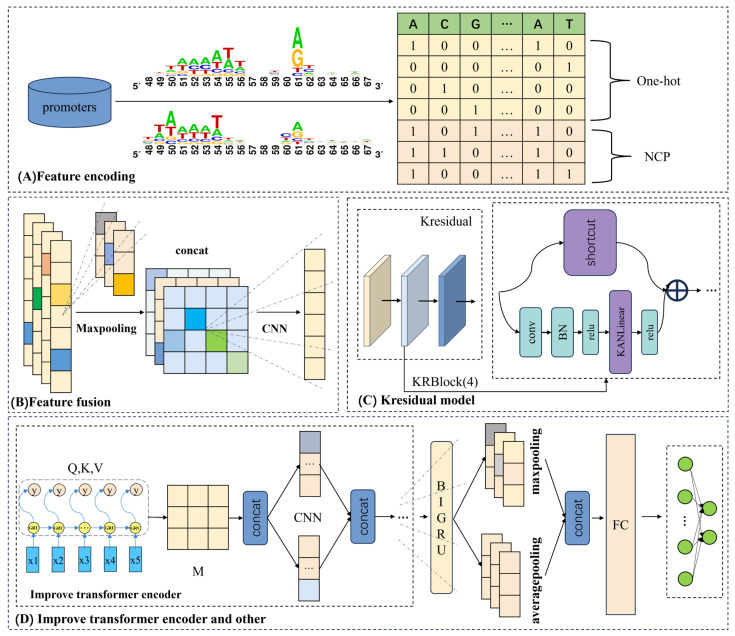
Model architecture diagram. (**A**) represents the feature representation section, implemented using one-hot and NCP encoding. (**B**) is the multi-scale convolution and pooling module. (**C**) is the Kresidual module. (**D**) shows the improved Transformer encoder module and other components.

**Figure 2:**
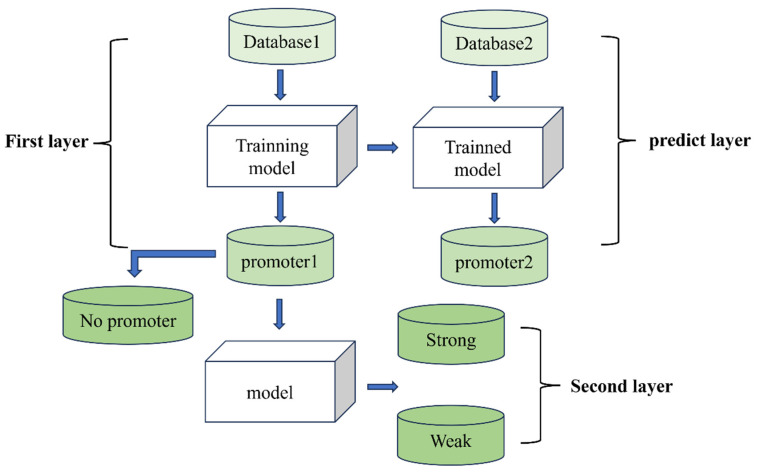
Overall flowchart.

The overall framework diagram in Figure 1 illustrates the approach adopted in this study, while the flowchart in Figure 2 presents the complete experimental procedure. The overall design of this study consists of the following three main steps: (i) constructing an effective benchmark dataset; (ii) introducing the framework of the proposed iPro2L-Kresidual model; and (iii) evaluating the predictive performance of the model through cross-validation.

The complete experimental procedure is shown in Figure 2. In Figure 2, Database1 represents the benchmark dataset, while Database2 is the independent dataset. First, the benchmark dataset is used to train the iPro2L-Kresidual model in the first stage, classifying sequences into “promoter” (promoter1) and “non-promoter” (No promoter) categories. Next, the trained iPro2L-Kresidual model from the first stage is used to predict and classify sequences in the independent dataset. Finally, the iPro2L-Kresidual model is applied for the second-stage training on the sequences identified as “promoter” (promoter1), further classifying them into “strong promoter” and “weak promoter” categories.

### 2.1. Benchmark Dataset

Selecting an appropriate and high-quality benchmark dataset is a crucial step for training and testing models. In this study, We used the benchmark dataset created by Xiao et al. [20]. They extracted promoter fragments from RegulonDB (version 9.4) [21] and experimentally verified that a reasonable promoter sequence length is 81 base pairs. In addition, they used CD-HIT [22] to remove promoter sequences with similarity exceeding 85%. Ultimately, The benchmark dataset consists of 3382 promoter sequences and 3382 non-promoter sequences. Among the promoter sequences, there are 1791 weak promoters and 1591 strong promoters, with promoter strength determined by their levels of transcriptional activation and expression. We adopted five-fold cross-validation for model training, where the dataset is divided into five parts, with each part being used as the validation set once and the remaining four parts for training the model. Furthermore, since the promoter dataset proposed by Xiao et al. lacks an independent test set, we chose to test on the E. coli promoter dataset proposed by Liu et al.

### 2.2. Feature Encoding

As shown in Figure 1A, we use hybrid encoding of one-hot and Nucleotide Chemical Property (NCP) to represent promoter sequences. One hot encoding only uses 0 and 1 to label nucleotide positions (e.g., A = 1000, T = 0100), and it completely ignores chemical properties of nucleotides. NCP encoding uses numerical values to characterize chemical properties, but cannot clearly label the specific position of nucleotides in the sequence. The hybrid coding used in this paper can leverage the respective advantages of two types of coding, which can characterize both the positional information and chemical properties of nucleotides. That is, the hybrid coding can accurately locate the position of nucleotides with specific chemical properties, avoiding functional misjudgment caused by positional confusion. The detail information of one-hot and NCP is as follows.

#### 2.2.1. One-Hot Encoding

One-hot encoding [23] is a simple and useful way to represent sequences, and it is often used in bioinformatics. In this study, we use this method to change the four building blocks of DNA into numbers. Each nucleotide (A, G, C, T) is identified by where the number ‘1’ is placed in a related list, which helps tell them apart. Nucleotide A is shown as (1, 0, 0, 0), T as (0, 1, 0, 0), C as (0, 0, 1, 0), and G as (0, 0, 0, 1). After using one-hot encoding, each DNA sequence is changed into a two-dimensional matrix that has n rows and 4 columns, where n is the length of the DNA sequence.

#### 2.2.2. NCP Encoding

Nucleotide Chemical Property (NCP) encoding [24] is a method that uses the chemical traits of nucleotides. This text talks about three important chemical features: ring shape, chemical groups, and strength of hydrogen bonds. First, adenine (A) and guanine (G) each have two ring shapes, while cytosine (C) and thymine (T) have just one. Second, adenine (A) and cytosine (C) have amino groups, but guanine (G) and thymine (T) have keto groups. Third, in the DNA double helix, adenine (A) pairs with thymine (T) by forming two hydrogen bonds, while cytosine (C) pairs with guanine (G) with three hydrogen bonds. By looking at these chemical features, a special code is created for each nucleotide, which is useful for many studies in bioinformatics.

Moreover, based on the above chemical characteristics, the nucleotide at position *i* in a DNA sequence can be represented as $N_{i}\left(R_{i},F_{i},H_{i}\right)$, where $R_{i},F_{i},H_{i}$, represent the three chemical features mentioned above. The equations are defined as follows:

$$R_{i}=\left\{\begin{array}{c} 1,P_{i}\in \left(A,G\right)\\ 0,P_{i}\in \left(C,T\right) \end{array}\right.\;\;\;\;\;\;F_{i}=\left\{\begin{array}{c} 1,P_{i}\in \left(A,C\right)\\ 0,P_{i}\in \left(G,T\right) \end{array}\right.\;\;\;\;\;H_{i}=\left\{\begin{array}{c} 1,P_{i}\in \left(A,T\right)\\ 0,P_{i}\in \left(C,G\right) \end{array}\right.\;\;$$

So, nucleotide A is shown as (1,1,1), nucleotide C as (0,1,0), nucleotide G as (1,0,0), and nucleotide T as (0,0,1). After NCP encoding, each DNA sequence will be turned into a two-dimensional grid that has n rows and 3 columns, where n is the length of the DNA sequence.

### 2.3. Model Architecture

#### 2.3.1. Multi-Scale Convolution and Pooling Module

The purpose of feature fusion is to integrate features from different sources or layers in order to enhance the richness and effectiveness of the feature set. Given the complexity of promoter sequence characteristics, this study aims to extract as much informative content as possible from sequence encoding, employing a multi-scale convolutional network as the primary feature extraction tool. To avoid insufficient feature extraction caused by using a single kernel size, four parallel one-dimensional convolutional layers with kernel sizes of 3, 5, 7, and 9 are designed to capture local features at various scales within the sequences. Additionally, each convolutional layer uses a different number of kernels to better accommodate the needs of multi-scale feature extraction.

To improve training efficiency and eliminate redundant features, the outputs from these four convolutional layers are integrated to form a comprehensive multi-scale feature map, which is then subjected to batch normalization and max pooling. Batch normalization effectively mitigates the interdependence among parameters during the update process, while max pooling reduces the dimensionality of the feature map through downsampling. Subsequently, a one-dimensional convolutional layer with a kernel size of 3 and 128 filters is applied to compress the number of channels in the feature map to 128, and the ReLU activation function is used to further enhance the feature representation. This series of operations not only reduces model complexity but also enables the efficient learning of nonlinear patterns within sequences, allowing the model to more effectively identify and extract complex biological features, thereby providing richer and more precise feature inputs for subsequent analysis and prediction [25].

#### 2.3.2. Kresidual Module

**Figure 3:**
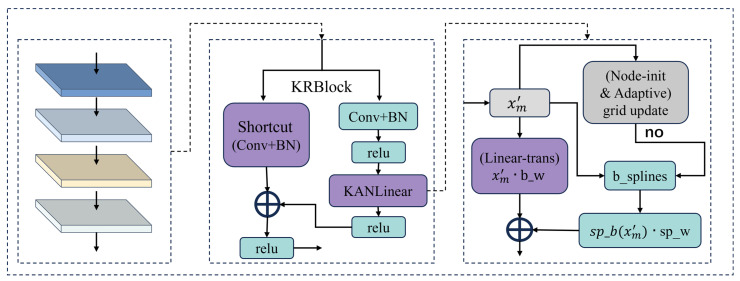
Kresidual module diagram.

ResNet, when implemented as a stack of deep MLPs or CNNs, lacks sensitivity to sequence structure when processing promoter sequences, resulting in weak interpretability with respect to biological characteristics. To address the insufficient nonlinear feature extraction capability of ResNet in promoter sequence tasks, this study specifically designs the Kresidual module, which deeply integrates the concepts of KAN (Kolmogorov–Arnold Network) [26,27] with the ResNet architecture [28], as shown in Figure 3. This integration endows the module with powerful nonlinear mapping capabilities while also improving training stability.

Specifically, the features $x_{m}$ generated by the multi-scale convolution and pooling module are used as the input to the Kresidual module. First, at the beginning of the main branch, $x_{m}$ is sequentially processed by a one-dimensional convolution, batch normalization, and a ReLU activation layer. The one-dimensional convolution is used to extract short-range correlations between local base pairs in $x_{m}$, batch normalization improves the stability of feature distributions, and ReLU activation enhances the model’s nonlinear expression capability. Through this hierarchical processing, $x_{m}$ is transformed into a feature representation with more prominent local dependencies and initial nonlinear characteristics, denoted as $x_{m}^{\prime }$.

Next, to address the limited nonlinear expressiveness caused by traditional fixed activation functions and to further enhance the network’s capacity for modeling nonlinear structures and achieving higher-order feature representation, we add a KANLinear layer to the end of the main branch. The transformed feature $x_{m}^{\prime }$ is then fed into the KANLinear layer. Unlike directly reusing the original KAN architecture, this study draws inspiration from the Kolmogorov–Arnold representation theorem (as shown in the following equation):

(1) $$F\left(x_{1},\ldots ,x_{n})\right.=\sum_{q=1}^{2n+1}\Phi_{q}\left(\sum_{p=1}^{n}\phi_{q,p}\left(x_{p}\right)\right)$$

That is, After inputting $x_{m}^{\prime }$ as the vectorized promoter sequence, it is nonlinearly mapped through the B-spline basis function $\phi_{q,p}(x_{p})$, decomposing the features into multiple segmented B-spline functions, each capturing the nonlinear relationship within a local feature interval. Compared with convolutional neural networks (CNNs) and multi-layer perceptrons (MLPs), they lack the ability to extract multiple local features and have difficulty modeling the correlations in sequences. However, the piecewise nonlinear modeling of B-splines can more flexibly capture multiple local feature patterns. The KANLinear layer first flattens the input into a two-dimensional representation, performs a basic linear transformation and B-spline nonlinear mapping, combines the two, and finally outputs the result:

(2) $${output}_{x}\;=x_{m}^{\prime }\cdot \mathrm{ase}\_\mathrm{weight}+spline_{bases\left(x_{m}^{\prime }\right)}\cdot spline_{weight}$$

Here, $spline\_bases(x_{m}^{\prime })$ denotes the basic linear weight matrix, while $spline\_bases(x_{m}^{\prime })$ represents the feature representations obtained by mapping the input xm′x_m’xm′ through B-spline basis functions. spline_weight refers to the weight matrix of the nonlinear B-spline component. This “decomposition–mapping–recombination” approach overcomes the limitations of single activation functions, enabling the extraction of richer high-order nonlinear features and greatly enhancing the model’s ability to capture complex relationships within sequences.

Secondly, to ensure that the network maintains efficient learning ability when faced with promoter sequences of different distributions, the KANLinear layer in this study automatically updates the positions of the B-spline basis function nodes according to the input features after each batch. In other words, after each input, the base nodes are dynamically adjusted based on the current sample distribution:

(3) $$\mathrm{grid}\;=\;0.02\;\times \;{\mathrm{grid}}_{\mathrm{uniform}}\;+\;0.98\;\times \;{\mathrm{grid}}_{\mathrm{adaptive}}$$

Here, grid refers to the positions of the B-spline basis function nodes. ${\mathrm{grid}}_{\mathrm{uniform}}$ indicates globally uniform distribution of the nodes, while ${\mathrm{grid}}_{\mathrm{adaptive}}$ denotes adaptive sampling of node positions based on the current batch feature distribution. This mechanism significantly enhances the network’s adaptability to data distribution shifts and improves its ability to fit higher-order nonlinearities.

In addition, in the design of the residual branch, to ensure strict alignment between the outputs of the main path and the KAN path in both channel and spatial dimensions, each residual branch of the KRANBlock uses a 1 × 1 convolution and BatchNorm to perform linear mapping on the input $x_{m}$:

(4) $${output}_{shortcut}=BN\left({Conv1D}_{1\times 1}\left(x_{m}\right)\right)$$

This not only enables lossless fusion of information between different feature paths, but also effectively avoids common issues such as gradient vanishing and feature degradation in deep models, providing strong support for feature aggregation in subsequent layers.

Finally, to further enhance the model’s ability to hierarchically abstract complex nonlinear structures, we stacked multiple KRANBlock modules in series. The output of each module, ${output}_{Kresidual}$, is activated by ReLU, progressively building a higher-level and more abstract feature space:

(5) $${output}_{Kresidual}=\sum_{i=1}^{4}relu\left(relu\left({output}_{x}^{\left(i\right)}\right)+{output}_{shortcut}^{\left(i\right)}\right)$$

Through this end-to-end hierarchical abstraction structure, the model is able to progressively integrate long-range dependencies and multi-scale information, maintaining the expressive power of a deep architecture while fully extracting nonlinear features.

In summary, the stepwise innovative design of the Kresidual module effectively addresses the shortcomings of traditional ResNet in extracting nonlinear features from promoter sequences, thereby providing richer and more precise feature inputs for subsequent analysis and prediction.

#### 2.3.3. Improved Transformer Encoder Module

When processing promoter sequence tasks, the GRU [29] excels at capturing local dependencies and sequential patterns within sequences, but lacks the ability to model long-range global dependencies, making it difficult to fully capture relationships between distant positions in the sequence. On the other hand, the Transformer encoder [30] is effective at capturing global dependencies, but is relatively weak in modeling local details and short-term dependencies in promoter sequences. This limitation is especially apparent when dealing with sequences that have subtle differences, as the Transformer encoder cannot fully capture the details of local variations. Therefore, to address the issue of insufficient capture of contextual relationships in promoter sequences, this study structurally reconstructs the multi-head attention module of the traditional Transformer encoder. Additionally, to enhance the modeling of both local and global features in promoter sequences and improve the model’s translational invariance, parallel convolutional processing is applied after the reconstructed multi-head attention module.

Specifically, the features generated by the previous module are first used as inputs to the improved Transformer encoder module. Unlike the traditional approach, instead of simply feeding the features into a standard multi-head attention module, we reprocess the Query (Q), Key (K), and Value (V) for each head. In particular, the conventional linear transformations are replaced with gated recurrent units (GRUs), enabling the model to capture dependencies more efficiently. The input is denoted as $Q_{pre}$, $K_{pre}$, $V_{pre}$. This modification allows the model to fully leverage the GRU’s ability to enhance the capture of both short-term and long-term dependencies when extracting features from promoter sequences, resulting in more refined representations for each head. Subsequently, the model models global dependencies by computing attention weights, as detailed below:

$$h_{t}^{Q_{pre}}=Z_{t}^{Q_{pre}}⊙h_{t-1}^{Q_{pre}}+\left(1-Z_{t}^{Q_{pre}}\right)⊙{\tilde{h}}_{t}^{Q_{pre}}$$

(6) $$h_{t}^{K_{pre}}=Z_{t}^{K_{pre}}⊙h_{t-1}^{K_{pre}}+\left(1-Z_{t}^{K_{pre}}\right)⊙{\tilde{h}}_{t}^{K_{pre}}$$

$$h_{t}^{V_{pre}}=Z_{t}^{V_{pre}}⊙h_{t-1}^{V_{pre}}+\left(1-Z_{t}^{V_{pre}}\right)⊙{\tilde{h}}_{t}^{V_{pre}}$$

First, update the $Q_{pre}$, $K_{pre}$, $V_{pre}$ at each position through GRU to obtain $h_{t}^{Q_{pre}}$, $h_{t}^{K_{pre}}$, and $h_{t}^{V_{pre}}$. Then, Calculate the attention weight scores using the $h_{t}^{Q_{pre}}$ and $h_{t}^{K_{pre}}$ features extracted by GRU. To facilitate the distinction of the $h_{t}^{Q_{pre}}$, $h_{t}^{K_{pre}}$, and $h_{t}^{V_{pre}}$ features at each position after the GRU update, we rename them accordingly as $h_{i}^{Q_{pre}}$, $h_{j}^{K_{pre}}$:

(7) $${Scores}_{i,j}=\frac{h_{i}^{Q_{pre}}\cdot {\left(h_{j}^{K_{pre}}\right)}^{T}}{\sqrt{d_{K_{pre}}}}$$

Generate the output of each head by weighting the $h_{j}^{V_{pre}}$ features extracted by GRU with the attention weights, where in $seq_{len}$ represents sequence length:

(8) $$head=\sum_{j=1}^{seq_{len}}\frac{\exp\left({Scores}_{i,j}\right)}{\sum_{j=1}^{seq_{len}}\exp\left({Scores}_{i,j}\right)}\cdot h_{j}^{V_{pre}}$$

Secondly, after completing spatial feature extraction, we further optimized the feature fusion process. All features from each head are first concatenated to form a more comprehensive and diverse sequence feature representation, denoted as $M_{multi}$:

(9) $$M_{multi}=Concat\left({head}_{1},\ldots ,{head}_{s}\right)$$

Next, the feature $M_{multi}$ is processed through parallel one-dimensional convolutional layers to further enhance the local feature extraction capability for promoter sequences. The convolution operation not only effectively captures local variations but also reduces computational complexity through dimensionality reduction. Subsequently, a linear layer is applied for further dimensionality reduction, avoiding excessive dimensionality caused by convolution operations.

Finally, to enhance the stability and generalization ability of the model, all processed features undergo layer normalization and other standard steps of the Transformer encoder, ensuring that the final output features are well-normalized. This series of structural adjustments represents a thorough reconstruction and redesign of the original module, aiming to extract both local and global features from sequences from multiple perspectives.

Therefore, through this multi-level feature extraction and refined module reconstruction, the model is able to capture and analyze various patterns in the input data with greater precision. This design not only enhances the feature modeling capability for promoter sequences but also provides strong feature support for subsequent prediction tasks. As a result of this systematic approach, the model’s accuracy and robustness in promoter sequence tasks are significantly improved. The algorithm for this module is presented below:
**Algorithm 1.** Procedure: Improve Transformer encoder1: procedure GRU_MultiHeadAttention_Conv(*x*, T) 2:   for each time step t in input sequence T do 3:     for forward pass in GRU layer do 4:       *fh*_t_ ← GRU(*x*_t_, *fh*_t__−__1_) 5:     for backward pass in GRU layer do 6:       *bh*_t_ ← GRU(*x*_t_, *bh*_t__+__1_) 7:     for each time step t do 8:       *h*_t_ ← *W*_1_ ·
*fh*_t_ + *V*_1_ ·
*bh*_t_ + *b*_1_ 9:   for each MultiHeadAttention layer do 10:     for each position t in sequence do 11:       Z_t_ ← MultiHeadAttention(*h*_t_) 12:       Pass through convolution submodule: 13:         Z_t_ ← Conv(Z_t_) + Conv(Z_t_) 14:         Z_t_ ← Linear(Z_t_) 15:       *h*_t_ ← LayerNorm(Z_t_ + FeedForward(Z_t_)) 16:       output H_1_ ← {*h*_1_, *h*_2_, ..., *h*_t_} 17:   for each time step t in H_1_ do 18:     for forward pass in GRU layer do 19:       *fh*_t_ ← GRU(*h*_t_, *fh*_t__−__1_) 20:     for backward pass in GRU layer do 21:       *bh*_t_ ← GRU(*h*_t_, *bh*_t__+__1_) 22:     for each time step t do 23:       *h*_t_ ← *W*_3_ 
·
*fh*_t_ + *V*_3_ ·
*bh*_t_ + *b*_3_ 24:   return H_2_ ← {*h*_1_, *h*_2_, ..., *h*_t_}

#### 2.3.4. Regularized Label Smoothing Cross-Entropy Loss Function

In the tasks of promoter identification and strength prediction, the traditional cross-entropy loss function has several limitations: it ignores the similarity between promoter classes, leads to instability during model training, and often results in overconfident predictions. For multi-class problems, its expression is as follows:

(10) $$L_{CE}=-\sum_{i=1}^{K}y_{i}\log\left({\hat{y}}_{i}\right)$$

Here, ${\hat{y}}_{i}$ denotes the true label encoding, $y_{i}\;$represents the predicted probability for class *i* by the model, and K is the total number of classes. To alleviate the model’s overconfidence in a single class and improve generalization ability, a label smoothing mechanism is introduced in this study. Specifically, we modify $L_{CE}$ by introducing a label smoothing parameter, changing the label distribution from “hard labels” to “soft labels”. The target class label is smoothed from 1 to 1−ϵ and non-target classes from 0 to ϵ. For the promoter classification task, the improved label smoothing cross-entropy loss function is expressed as follows:

(11) $$L_{2_{smooth}}=-\left[\left(1-\epsilon \right)\cdot y\cdot \log\left(\hat{y}\right)+\epsilon \cdot \left(1-y\right)\cdot \log\left(1-\hat{y}\right)\right]$$

Here, ϵ is the label smoothing factor, y is the true label, and $\hat{y}$ is the predicted probability from the model. This approach effectively suppresses overconfidence in a single class, enhances the model’s robustness to sample perturbations, and thus improves generalization ability.

On this basis, to further enhance the stability of model training, an $L_{2}$ distribution regularization term on the predicted probability $\hat{y}$ is introduced into the label smoothing cross-entropy loss function. The final loss function is expressed as follows:

(12) $$L_{reg-smooth}=L_{2_{smooth}}+\lambda \cdot {\left|\left|\hat{y}\right|\right|}_{2}^{2}$$

Here, λ is the regularization coefficient, and ${\left|\left|\hat{y}\right|\right|}_{2}^{2}$ denotes the squared $L_{2}$-norm of the model’s output probability distribution. With these two improvements, the loss function used in this study retains the advantages of the original cross-entropy loss while effectively optimizing the label distribution and loss structure, thus providing a solid foundation for subsequent model training.

### 2.4. Model Training

In this study, the iPro2L-Kresidual model was implemented using the TensorFlow and Keras frameworks and trained on an NVIDIA GeForce RTX 1050i GPU. To ensure fairness in performance comparison, we adopted five-fold cross-validation, which is consistent with previous studies. To prevent overfitting and model overconfidence, we employed a regularized label smoothing cross-entropy loss function.

For optimization, we used the Adam algorithm [31], which is widely adopted due to its adaptive learning rate adjustment capabilities. To further mitigate overfitting, several strategies were applied: in terms of activation functions, we used the ReLU function. For regularization, batch normalization was implemented to stabilize learning by adjusting the scale of input data, thereby reducing dependence on specific training samples. In addition, dropout layers were applied to randomly deactivate a portion of neurons during training, reducing co-adaptation among neurons and improving generalization.

These techniques together ensure that the model maintains strong expressive power while effectively avoiding overfitting and improving generalization performance. Moreover, we employed early stopping: if the model’s loss function ceased to decrease significantly or met a predefined threshold, training would be terminated early to avoid unnecessary computational cost. For parameter optimization, we carefully tuned the model based on findings from prior research [32].

### 2.5. Evaluation Metrics

Scientific evaluation criteria are essential for assessing models. In this experiment, we adopt the evaluation metrics commonly used by previous researchers, including sensitivity (Sn), specificity (Sp), accuracy (Acc), and Matthews correlation coefficient (MCC) [33]. Sensitivity and specificity evaluate the model’s recognition ability from the perspectives of promoters, non-promoters, strong promoters, and weak promoters. Accuracy provides an intuitive measure of the overall classification performance, while the Matthews correlation coefficient comprehensively considers the model’s two-stage classification capability, making it particularly suitable for handling imbalanced datasets. In this study, these metrics are calculated and analyzed to provide a multidimensional assessment of model performance, thereby offering a more reliable basis for our research conclusions. By comprehensively analyzing these four metrics, the overall performance of the model or diagnostic method can be thoroughly evaluated. The calculation formulas are as follows:

(13) $$SP=\frac{TN}{TN+FP}$$

(14) $$SN=\frac{TP}{TP+FN}$$

(15) $$ACC=\frac{TP+TN}{TP+FN+TN+FP}$$

(16) $$MCC=\frac{\left(TP\times TN\right)-\left(FP\times FN\right)}{\sqrt{\left(TP+FP\right)\left(TP+FN\right)\left(TN+FP\right)\left(TN+FN\right)}}$$

In addition, the confusion matrix is an indispensable tool in the calculation of these evaluation metrics, playing a key role especially in model performance evaluation and binary classification tasks. By comparing the predicted results with the actual outcomes, the confusion matrix intuitively displays the model’s classification performance in matrix form. The key performance indicators in the confusion matrix include: true positives (TPs), true negatives (TNs), false positives (FPs), and false negatives (FNs) [34].

## 3. Results and Discussion

This section consists of three parts: (i) Ablation experiments on the main components of iPro2L-Kresidual are conducted by replacing some modules and performing ablation analysis on the hyperparameters of the main modules. The model’s performance under different structures and parameter configurations is compared to demonstrate the impact of the main modules and parameter settings on model performance. (ii) 5-fold cross-validation performance evaluation of iPro2L-Kresidual. (iii) Finally, a comparison is made with the current state-of-the-art models on 5-fold cross-validation.

### 3.1. Ablation Experiments

To systematically evaluate the effectiveness of the proposed improved Transformer encoder and Kresidual structure, this paper designs the following five ablation experiments: (i) Compare the performance differences between Kresidual and traditional residual connections in the promoter identification task; (ii) Analyze the impact of the number of Kresidual residual blocks on the performance of the promoter identification and strength classification tasks; (iii) Compare the performance of the basic Transformer encoder and the improved Transformer encoder in the promoter identification and strength classification tasks, in order to verify the advancement and effectiveness of the improved Transformer encoder; (iv) Investigate the impact of the number of encoder blocks on the improved Transformer encoder based on the performance of the promoter identification and strength classification tasks; (v) Explore the impact of the number of attention heads on the improved Transformer encoder based on the performance of the promoter identification and strength classification tasks.

**Figure 4:**
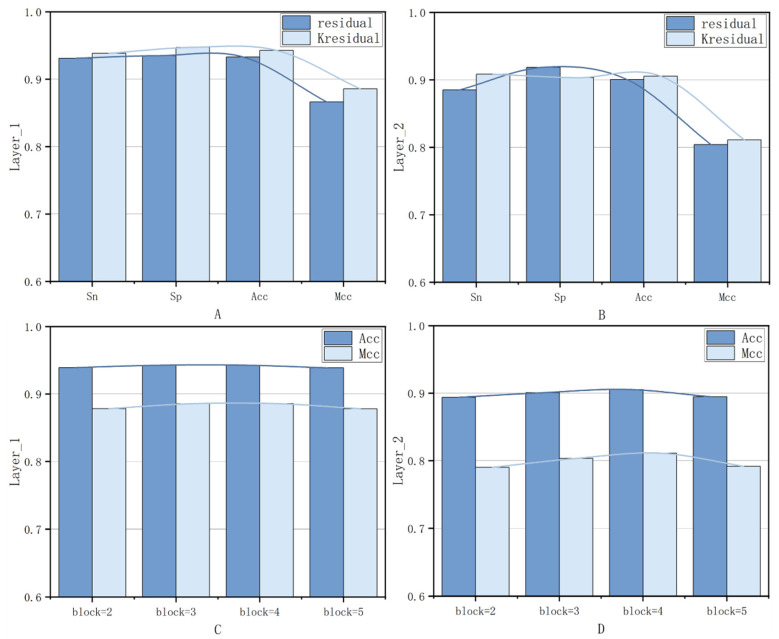
Experimental results of different residual networks, and the performance of Kresidual module varying with the number of residual blocks. (**A**,**B**): the comparison of Sn, Sp, Acc, and MCC curves for different residual networks in the first and second layers, respectively. (**C**,**D**): the comparison of Acc and MCC curves for the Kresidual module varying with the numbers of residual blocks in the first and second layers, respectively.

To systematically evaluate the effects of the five factors mentioned above in the promoter identification and strength classification tasks, this paper conducts the analysis while keeping the parameters of other modules constant. First, a comparison experiment between the residual and Kresidual structures was carried out, and the experimental results are shown in Table 1 and Figure 4. As shown in Figure 4A, in the promoter identification task (First layer), after applying the Kresidual structure, the model’s ACC and MCC improved by 0.98% and 1.95%, respectively. As shown in Figure 4B, in the promoter strength classification task (Second layer), the ACC and MCC improved by 0.51% and 0.70%, respectively. These results indicate that the Kresidual structure significantly enhances the performance of the two-stage promoter classification model.

Following the same experimental methodology, the second ablation experiment was validated. As shown in Figure 4C,D, when comparing the performance with different numbers of Kresidual residual blocks, the performance of the two-stage promoter classification model was optimal when the number of blocks was 4.

Subsequently, the third ablation experiment was conducted for a comparative analysis to evaluate the advantages of the improved Transformer encoder over the basic Transformer encoder. As shown in Table 2, the improved Transformer encoder increased the ACC and MCC by 0.93% and 1.69%, respectively, in the promoter identification task (First layer). In the promoter strength classification task (Second layer), the ACC and MCC improved by 0.57% and 1.14%, respectively.

**Figure 5:**
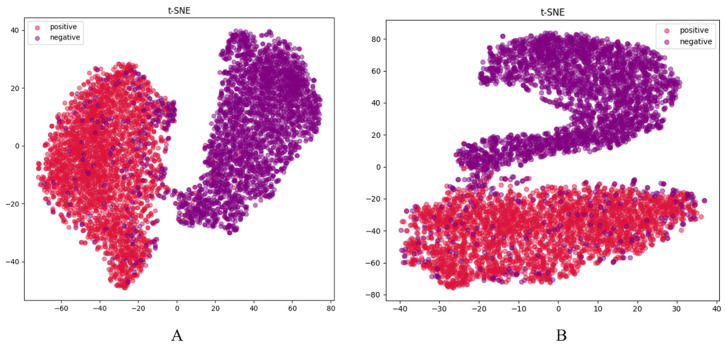
t-SNE visualization results of different Transformer encoder. (**A**) the improved Transformer encoder; (**B**) the original Transformer encoder.

Additionally, in the model generalization capability experiment, the t-SNE visualization results (Figure 5) show a more distinct separation between positive and negative samples in the feature space. Figure 5A corresponds to the improved Transformer encoder module, while Figure 5B corresponds to the unmodified module. A comparison shows that the improved module performs significantly better in terms of classification effectiveness. Therefore, the improved Transformer encoder demonstrates superior applicability and generalization ability in this task.

To further investigate the improved Transformer encoder module, the ablation experiment was conducted. This experiment evaluated the effect of different values for the encoder count (2, 3, 4, 5) on the improved Transformer encoder. As shown in Table 3, in the promoter recognition task and intensity classification task, when the count is 4, the accuracy (ACC) and Matthews correlation coefficient (MCC) reach 94.28% and 88.58%, 90.55% and 81.12%, respectively, which are superior to the performance when the count is 2, 3 and 5. Therefore, in this paper, we set encoder count to 4.

**Figure 6:**
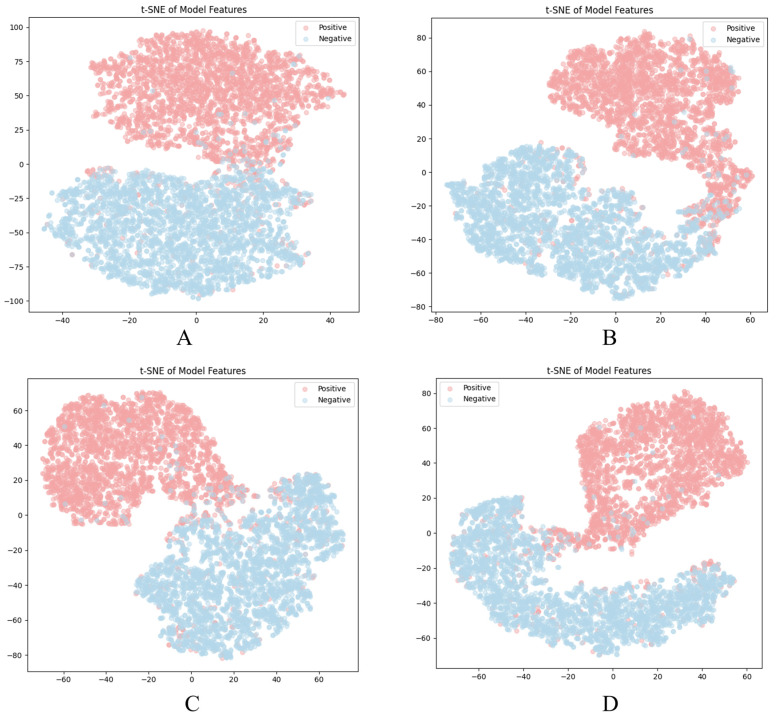
t-SNE visualization results of different encoder counts on an independent dataset (Note: Figures (**A**–**D**) correspond to counts of 2, 3, 4, and 5, respectively).

Additionally, t-SNE visualization results of different encoder counts on an independent dataset are shown in Figure 6. When the count was 4, the distinction between the two clusters (positive, negative) in the t-SNE plot was most prominent, as shown in Figure 6C.

**Figure 7:**
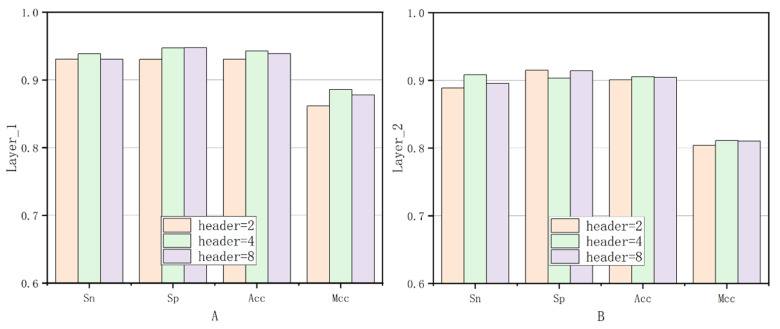
Experimental comparison of different headers on Layer1 and Layer2.

Finally, based on the optimal encoder count, the fifth ablation experiment was conducted to compare the performance with different numbers of heads in the multi-head attention mechanism. As shown in Figure 7, the module’s performance was optimal when the number of heads (header) was 4. In Figure 7A, the performance comparison of Sn, Sp, ACC, and MCC is shown for header values of 2, 4, and 8 in the first layer. Figure 7B presents a similar comparison for the second layer.

### 3.2. K-Fold Cross-Validation

Cross-validation provides a powerful and effective method for evaluating a model using the entire dataset. It divides the data into k subsets or folds, where each fold serves as the test set once while the remaining k-1folds are used for training. In this study, k is set to 5, so the dataset is divided into five folds. Each cross-validation cycle involves training on four folds and testing on the remaining one fold. This process is repeated ten times to comprehensively assess the model’s performance. This approach helps evaluate the model’s effectiveness across various segments of the data and assesses its stability.

**Figure 8:**
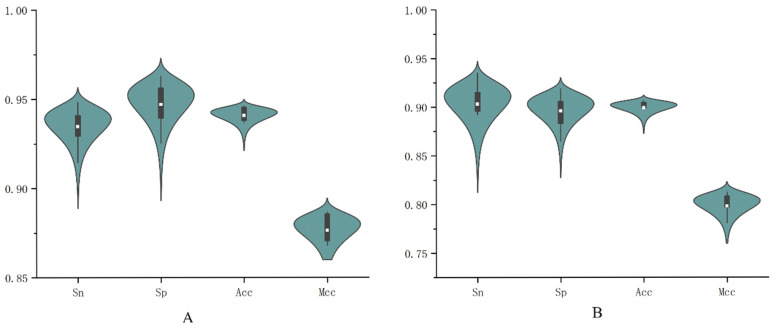
The results of ten runs of five-fold cross-validation for the two-stage classification of promoters ((**A**) for promoter identification, (**B**) for promoter strength identification).

The evaluation results are shown in Figure 8, where Figure 8A,B represent the first-stage and second-stage promoter classification, respectively. From the shape of the violin plot in Figure 8A, the distribution of accuracy is relatively concentrated, indicating that the model’s accuracy is quite stable across 10 runs of five-fold cross-validation. The average value is approximately 0.94, suggesting that the model can achieve around 0.94 accuracy in most cases and performs very well in correctly classifying samples. The generally high values and absence of significant outliers in the distribution further demonstrate that the model delivers consistent performance across different data splits, reflecting strong generalization ability. The same pattern is observed in Figure 8B. These results show that the model achieves excellent performance on the current dataset, exhibiting high accuracy, good balance, and robustness.

### 3.3. Comparison with Existing Methods Based on the Benchmark Dataset

Over time, an increasing number of machine learning and deep learning methods have been applied to the field of promoters. To evaluate the performance of the iPro2L-Kresidual predictor, we compare it with five other outstanding predictors. This comparison is based on the same dataset and validation methods. When comparing the performance of the iPro2L-Kresidual predictor with several other predictors, iPro2L-Kresidual outperforms the others across four key evaluation metrics: Sensitivity (Sn), Specificity (Sp), Accuracy (Acc), and Matthews Correlation Coefficient (MCC).

In the analysis of other predictors, we observed that iPSW(2L)-PseKNC, although using machine learning methods, did not perform well in terms of prediction accuracy. On the other hand, iPSW(PseDNC-DL) [35] and FastText N-grams [36] are predictors based on Convolutional Neural Networks (CNNs). Despite the tremendous success of CNNs in areas like image recognition, traditional CNNs may not fully unleash their potential in sequence analysis tasks due to insufficient feature extraction capabilities. This could be one of the reasons for the poor performance of these models. Additionally, iPro2L-CLA is a predictor based on Capsule Networks. Capsule Networks were designed to address some inherent problems in CNNs, such as better handling of spatial hierarchies and viewpoint variations. However, Capsule Networks also have certain limitations in improving prediction performance and are unable to fully overcome the shortcomings of CNNs.

Overall, the iPro2L-Kresidual predictor outperforms several other predictors across numerous evaluation metrics. It not only demonstrates superiority and broad applicability in sequence analysis tasks but also eliminates certain performance shortcomings to varying degrees when compared to other predictors based on machine learning, CNNs, and Capsule Networks.

As shown in Table 4, in the promoter recognition task, our model is 2.42% higher in ACC compared to the newer iPro-TCN model. In the promoter strength classification task, our model outperforms by 5.96% in ACC. This comparison demonstrates that the iPro2L-Kresidual predictor performs well in the two-stage promoter prediction task. Notably, even in the second layer task, which has a smaller dataset, the predictor maintains relatively high prediction accuracy. This not only reflects the stability and excellent predictive performance of iPro2L-Kresidual in situations with limited data, but also suggests that, as the promoter dataset size increases, the predictive capability of iPro2L-Kresidual is expected to improve further.

In other words, the iPro2L-Kresidual predictor has achieved good results in both promoter recognition and strength classification tasks. Particularly in the second layer prediction, where the dataset is smaller, it still maintains high predictive accuracy. This indicates that even with limited data, the iPro2L-Kresidual predictor can maintain good consistency and superiority. Therefore, it is expected that as the available promoter data increases, the predictive performance of the iPro2L-Kresidual predictor will be further enhanced.

### 3.4. Comparison with Existing Methods Based on Independent Datasets

Additionally, considering the lack of an independent test set in the promoter dataset proposed by Xiao et al., we chose the *Escherichia coli* promoter dataset proposed by Liu et al. [37] to test the generalization ability of the iPro2L-Kresidual predictor. The test results, as shown in Table 5, indicate that our predictor performs better than most other predictors on the *E. coli* promoter dataset as well, Including PCSF [38], vw Z-curve [39], Stability [40], iPro54 [41], iPromoter-2L, iPSW(2L)-PseKNC, iPro2L-CLA, and iPro-TCN (without validation using an independent test set).

Therefore, based on these data, it can be concluded that the iPro2L-Kresidual predictor demonstrates good stability and generalization ability when predicting other types of promoter tasks. It is also one of the outstanding predictors in promoter prediction tasks.

## 4. Conclusions

In this study, we propose a deep learning-based “iPro2L-Kresidual” model to identify promoters and their strength. The advantage of this model lies in two key aspects. On the one hand, the integration of residual structures with the KAN network constructs the Kresidual module, compensating for the limitations of traditional methods in feature extraction. On the other hand, the improved Transformer encoder module captures both global and local features, addressing the issue of insufficient sequence context relationship mining. Additionally, to ensure the stability of model training and prevent the model from becoming overly confident, we designed a regularized label smoothing cross-entropy loss function. However, this model still has certain limitations. Due to the scarcity of promoter datasets, the batch size had to be set relatively small, specifically at 30. Moreover, the adaptive weight update in the Kresidual module has a specific dependency on the batch size. During the experiments, based on the observed phenomena at that time, enabling the adaptive node update would cause the network to fail to converge. Therefore, to ensure the model operates under stable convergence, we chose not to enable the adaptive node update in this experiment. Although this choice effectively avoided potential convergence issues, it might also have led to the model not fully utilizing its resources. Based on this experimental phenomenon, we speculate that the model’s performance might not have reached its optimal level. Future work will further verify this hypothesis and explore the potential of activating the adaptive node update under appropriate conditions to enhance the model’s performance.

Nonetheless, after extensive experimental validation, the iPro2L-Kresidual method performs well in two-stage promoter prediction. It not only accurately predicts whether a sequence is a promoter but also effectively assesses its strength. Compared to existing methods, it shows a significant improvement in performance.

Furthermore, since abnormal regulation of promoters is associated with various diseases, the two-stage classification of promoters presented in this study contributes to the development of biomedical research on disease mechanisms and gene therapy. Therefore, in future work, we plan to further expand the iPro2L-Kresidual method into the iPro2L-Kresidual model and deploy it on a server network. By constructing an easy-to-access and powerful online tool, researchers will be able to conveniently use the model for promoter prediction and analysis, providing more research opportunities and potential value for scientists in bioinformatics and biomedical fields. The development of this online tool will not only improve research efficiency but also promote collaboration and communication between different research teams, further advancing genomics and biomedical research.

## Figures and Tables

**Table 1:** Comparison between traditional residual networks and Kresidual.

Layer	Model	Sn	Sp	Acc	Mcc
First layer	residual	0.9311	0.9349	0.9330	0.8663
	Kresidual	0.9385	0.9473	**0.9428**	**0.8858**
Second layer	residual	0.8851	0.9187	0.9004	0.8042
	Kresidual	0.9084	0.9032	**0.9055**	**0.8112**

**Table 2:** Comparison of the original Transformer encoder and the improved Transformer encoder.

Layer	Model	Sn	Sp	Acc	Mcc
First layer	No improved	0.9219	0.9459	0.9335	0.8689
	Improved	**0.9385**	**0.9473**	**0.9428**	**0.8858**
Second layer	No improved	0.9259	0.8741	0.8998	0.7998
	Improved	0.9084	**0.9032**	**0.9055**	**0.8112**

**Table 3:** Comparison of the Improved Transformer Encoder with Different Encoder Counts.

Layer	Encoder Counts	Sn	Sp	Acc	Mcc
First layer	2	0.9164	0.9531	0.9341	0.8704
	3	0.9414	0.9388	0.9401	0.8804
	4	0.9385	0.9473	**0.9428**	**0.8858**
	5	0.9170	0.9545	0.9354	0.8727
Second layer	2	0.9232	0.8831	0.9035	0.8085
	3	0.9011	0.9086	0.9049	0.8099
	4	0.9084	0.9032	**0.9055**	**0.8112**
	5	0.8920	0.9154	0.9043	0.8089

**Table 4:** Comparison with existing prediction models.

Layer	Predictors	Sn	Sp	Acc	Mcc
First layer	iPSW(2L)-PseKNC	0.8137	0.8489	0.8313	0.6630
	iPSW(PseDNC-DL)	0.8334	0.8683	0.8510	0.7024
	FastText N-grams	0.8276	0.8805	0.8541	0.7090
	iPro2L-CLA	0.8687	0.8513	0.8600	0.7211
	iPro-TCN	0.9274	0.9100	0.9186	
	iPro2L-Kresidual	**0.9385**	**0.9473**	**0.9428**	**0.8858**
Second layer	iPSW(2L)-PseKNC	0.6223	0.7917	0.7120	0.4213
	iPSW(PseDNC-DL)	0.6581	0.7816	0.7235	0.4440
	FastText N-grams	0.6940	0.7640	0.7310	0.4600
	iPro2L-CLA	0.7763	0.6878	0.7346	0.4700
	iPro-TCN	0.8198	0.8704	0.8463	
	iPro2L-Kresidual	**0.9084**	**0.9032**	**0.9055**	**0.8112**

**Table 5:** Comparison on the benchmark dataset provided by Liu et al.

Method	Sn	Sp	Acc	Mcc
PCSF	0.7890	0.7070	0.7480	0.4980
vw Z-curve	0.7776	0.8280	0.8028	0.6100
Stability	0.7660	0.7950	0.7800	0.5620
iPro54	0.7776	0.8315	0.8045	0.6100
iPromoter-2L	0.7920	0.8416	0.8168	0.6343
iPSW(2L)-PseKNC	0.8378	0.8434	0.8406	0.6811
iPro2L-CLA	0.8627	0.8480	0.8553	0.7114
iPro2L-Kresidual	**0.9885**	**0.8741**	**0.9313**	**0.8683**

## Data Availability

The data set and source code used in this study can be easily derived from https://github.com/lsc-csl/iPro2L-Kresidual (accessed on 14 November 2025).
